# Association of microstructural lesions of the corpus callosum with cognitive impairment in patients with high grade glioma

**DOI:** 10.1007/s00701-025-06467-x

**Published:** 2025-03-14

**Authors:** Xenia Hautmann, Carolin Weiss Lucas, Roland Goldbrunner, Mario Löhr, Gyoergy Homola, Ralf-Ingo Ernestus, Stefan Rueckriegel

**Affiliations:** 1 Department of Neurosurgery, University Hospital Wuerzburg, Josef-Schneider-Str. 11, Wuerzburg, 97080 Germany; 2 Department of Neuroradiology, University Hospital Wuerzburg, Wuerzburg, Germany; 3 Center of Neurosurgery, University Hospital of Cologne, Cologne, Germany

**Keywords:** Glioma, DTI, Neuropsychology, Corpus callosum, White matter integrity

## Abstract

**Purpose:**

Glioblastoma is one of the most common malignant brain tumors. To ensure a treatment that does not only lengthen survival, but also improves preservation of neurocognitive functions, reliable methods to measure changes in neurocognitive abilities at an early stage are necessary. The most direct way to objectify neurocognitive properties is neuropsychological testing. Neurocognitive decline is often based on lesions of the connectome. We take the corpus callosum (CC) as a reliable structure to identify decline of white matter (WM) integrity. We hypothesized a relation between compromised structural integrity in specific regions of the CC and neurocognitive deficits in glioma patients.

**Methods:**

We included 28 patients with high-grade glioma who underwent a neuropsychological test battery and MRI with Diffusion tensor imaging (DTI) preoperatively. MRI data was processed using the software fsl, Oxford. Neuropsychological parameters were correlated with the fractional anisotropy (FA) in three parts of the CC.

**Results:**

Preoperatively, most of the neuropsychological parameters correlated significantly with FA of at least one of the CC volumes. Higher FA-values were associated with better focus, memory, speed and speech fluency. Different tests examined the same neuropsychological parameter and then correlated with the same region of the CC.

**Conclusions:**

We consider the FA of the CC for an adequate parameter to examine the influence of distant lesions on neurocognitive abilities.

## Introduction

 Glioblastoma is the most common malignant brain tumor of glial origin in adults [37]. With a median survival of 15 months [45] the prognosis is still poor. Unfortunately, patients undergoing treatment do often develop a neurocognitive decline. Even before surgery, 75% of all glioblastoma patients do suffer from cognitive impairment [35], which has a high impact on their quality of life [16]. Survival time is often the only factor of treatment success in glioma patients [33], while quality of life plays a minor role. In consideration of the poor prognosis, quality of life should be more emphasized when evaluating treatment success.

To ensure a treatment that does not only lengthen survival, but also improves preservation of neurocognitive functions, risk factors for the development of neurocognitive deficits must be identified. A reliable method to measure changes in neurocognitive abilities at an early stage is needed in order to identify and prevent such risk factors in the future.

The most direct way to objectify neurocognitive abilities is neuropsychological performance testing. There is a variety of valid tests that examine different neuropsychological parameters. Nonetheless, scientific evidence concerning prevalence and course of neurocognitive impairment in glioma patients is poor. In 2003, Meyers and Hess declared that neuropsychological tests are usually sensitive to circumcised lesions and that a testing battery containing different tests must be used [33].

The multicenter NOA-19 study (under the lead of the University of Cologne) was based on this idea and defined the neuropsychological test battery used in this study [50]. The University Hospital Würzburg participated in this study.

Often, glioma patients are already in a poor state of cognition, which impedes neuropsychological testing. The patients’ current psychological condition and short-term treatment effects like fatigue often affect testing results. Therefore, the extension of our understanding of associated morphological changes of the connectome in this patient group could be very helpful. Microstructural lesions of the cerebral white matter (WM) are important morphological correlates to neurocognitive impairment [32, 15]. Accordingly, we hypothesized a relation between WM lesions in specific regions of the connectome and neuropsychological deficits in glioma patients.

Conventional MR-imaging is not sensitive enough to identify subtle white matter lesions. MR Diffusion tensor imaging (DTI) is more sensitive to classify compromised structural integrity in normal appearing WM [3].

Using DTI, we measured the compromised structural integrity in three parts of the corpus callosum (CC). As a central part of the WM and in consideration of the different tumor locations, we consider the CC to be an appropriate correlate of WM microstructure. With a maximum length of 90 mm, the CC is the largest fiber tract of the human brain, connecting both hemispheres and with it the different brain lobes [21]. Lesions of the CC are usually associated with neuropsychological deficits. Aim of this study is to prove an association between reduced FA and neuropsychological decline in glioma patients.

## Methods

### Patients

Adult patients with the initial diagnosis of a high grade glioma (WHO °III-IV) who were treated at the University Hospital Würzburg were included to this study. In- and exclusion criteria were defined by the NOA-19 study. Inclusion criteria were: tumors with a supratentorial, monolocular location in T1 weighted, contrast medium imaging, Karnofsky Index > 70% [41], at least 20 points in Mini Mental Status Test (MMST) [17]. Multilocular, bihemispheric and infratentorial tumor location as well as severe depression, preceded head radiation and chemotherapy during the last two years and insufficiently treated epilepsy were exclusion criteria.

### Neuropsychological testing

The neuropsychological test battery was defined by NOA-19 study [50]. The tests were performed using bed-side paper-pencil tests in a quiet room by a trained investigator using standardized instructions. Table 1 summarizes the elements of the test battery. Patients were examined preoperatively within one week before surgery. Some tests were modified to reduce patient’s burden.

**Table 1:** Neuropsychological tests, table taken from NOA-19 study synopsis and adapted [50]

Test	Implementation	References
Digit Span Test (DST)	Focus, verbal working memory *Repetition of numbers*	[49]
Symbol Digit Modality Test (SDMT)	Graphomotor speed and working memory *geometric symbols must be assigned to a number*	[47, 49]
Rey-Osterrieth Complex Figure Test (ROCF) I	Visuospatial abilities, executive functions *Copying of a geometric figure*	[39, 42]
Hopkins Verbal Learning Test-Revised (HVLMT-R) I	Verbal working memory *Repetition of 12 words*	[7]
Stroop ColorWord Test (SCWT)	Selective attention *Naming of color*s	[30, 44]
Trail Making Test (TMT)	Visuospatial attention, executive functions and motor skills *Connecting numbers with a line (pencil)*	[38]
Judgement of Line Orientation Test (JLOT)	Spatial orientation *Matching lines with numbers referenced to distinct line orientations*	[10]
9 Hole Pegboard Test (9-HPT)	Fine motor skills *Sorting rods in a board, and putting them back*	[48]
Controlled Oral Word Association Test (COWAT)	Lexical fluency of speech *Free association of words to an initial letter (consonant)*	[9, 40]
Symbol Cancellation Test (Find the Symbol Test; FST)	Visual comprehension *Symbols need to be marked*	[19]
Hopkins Verbal Learning Test-Rev. (HVLMT-R) II	Verbal memory Repetition and recognition of the 12 words from part I	[7]
Rey-Osterrieth Complex Figure Test (ROCF) II	Spatial and visual memory *Drawing a figure (copied in part I) from memory*	[36, 39, 42]

### MRI acquisition and processing

MR-imaging containing DTI was performed within one week before surgery on on a 3.0-T MRI system according to the clinical routine protocol for preoperative MRI of brain tumour patients with a 12- channel headcoil (MAGNETOM Trio [Siemens Healthcare, Erlangen, Germany]) using 64 diffusion (b-value: 1000) encoding directions at an in-plane resolution of 1.8 mm with slice thickness of 3.6 mm and averaging 2 preprocessed datasets, b-value 1000. T1-weighted magnetization prepared rapid gradient echo images were recorded with and without contrast enhancement. Repetition time (TR) was 2530 ms, echo time (TE) was 3.4 ms. The flip angle (FA) was 7°. Inversion recovery (IR) was 1200 ms and field of view (FOV) was 256 mm, matrix size was 256 × 256. DTI measures the diffusion of water molecules in the brain tissue. Since cerebral WM consists of axons, water molecules cannot move unimpeded, but are limited by cell membranes and myelin. Hence, they move mainly in the direction of fiber tracts. An important DTI parameter is fractional anisotropy (FA). It measures the predominance of one direction of diffusion. Accordingly, it decreases when neuronal tracts decay. A high FA-value implies a good functionality of neuronal tracts [24] and is an adequate measure for WM integrity.

**Fig. 1:**
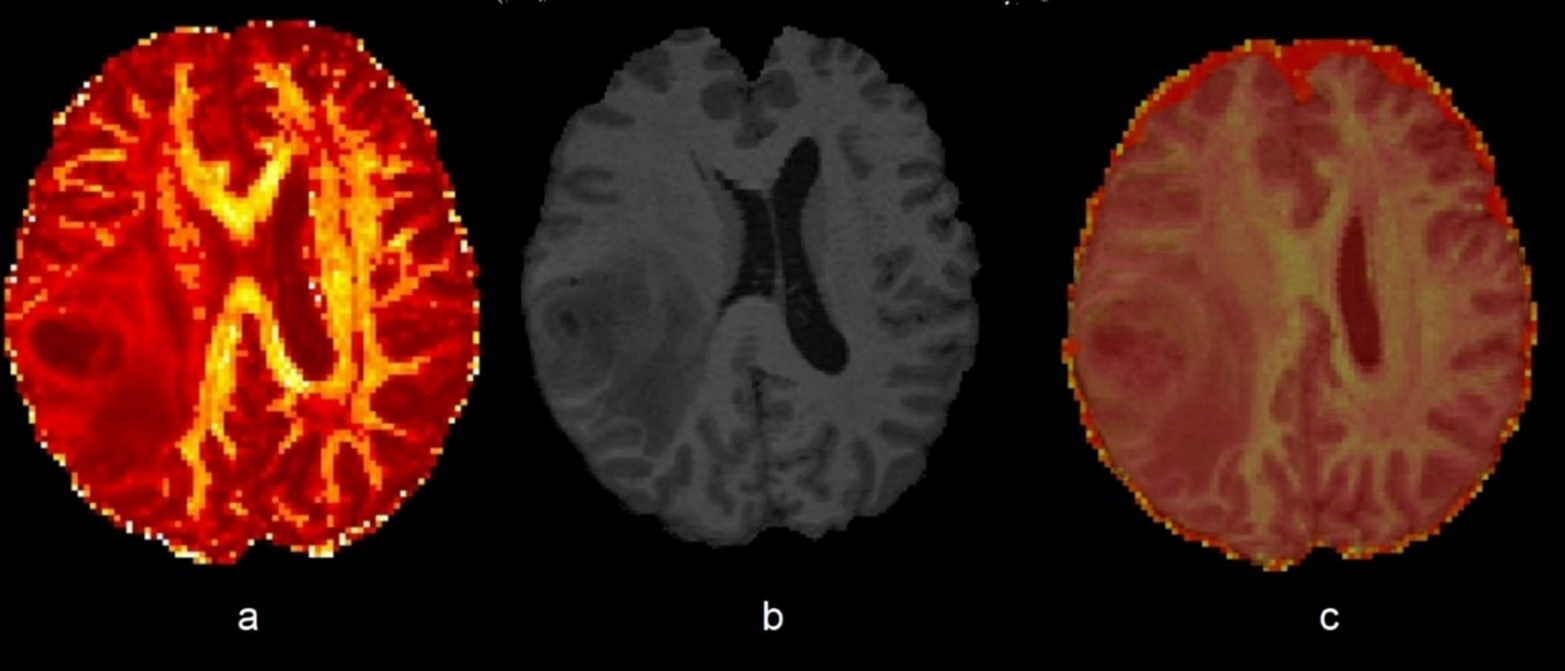
**a** FA-map, high FA values are yellow, low values are red, **b** processed T1 MRI , **c** fusion of MPRAGE T1 and FA-map

The aim of our MRI processing was to create a map containing all FA-values of the brain and to merge it with a high-resolution anatomical image to measure the mean FA in three parts of the CC. For processing we used the software fsl Oxford [27, 51]. We created the **FA map** based on Diffusion weighted imaging (DWI) sequences: The first step was “**eddy current correction**,** FSL**” [1, 2, 4] to correct artefacts and eddy currents by head movement. The next processing step was done using “**BET brain extraction**” [43, 25, 28], which removed non-brain tissue in the image as bone or skin. To prevent, that brain tissue is removed as well, the result was checked using “fsleyes”, a MRI viewer. Tensors and FA were then processed using the tool “**DTI Fit**” [5, 6, 23]. Then a structural MR image (T1 MPRAGE without contrast agent) was also processed using the tools “Eddy current correction” and “BET” [43, 28]. The last step of post-processing was done using “**FLIRT**”, a tool for linear image registration [26, 29, 22]. Figure 1 shows the FA map, the processed MPRAGE and the merged image containing both, anatomical and FA data. In accordance with the anatomical division of the CC in genu, truncus and splenium, we defined three cuboid volumes inside the CC. The following directions are saggital x axial x coronar:

- an anterior part “**CCA**” in the genu measures 3 × 3 × 3 voxel,
- a middle part “**CCM**” in the truncus measures 15 × 1 × 3 voxel and.
- a posterior part “**CCP**” in the splenium measures 3 × 3 × 3 voxel.

**Fig. 2:**
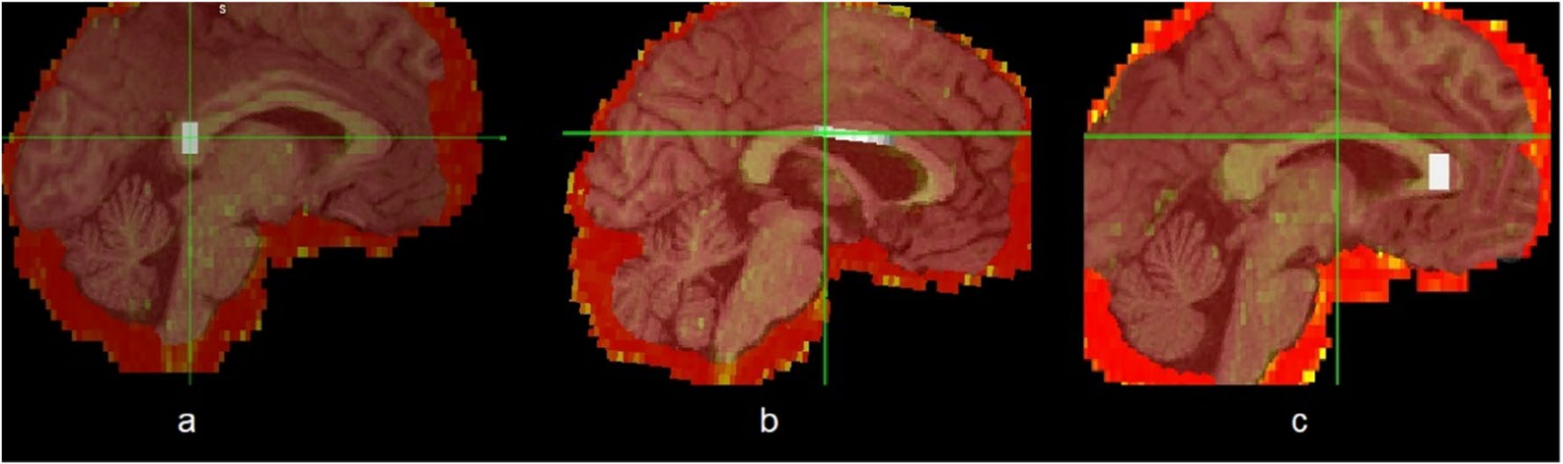
Volumes rendered to fused image (FA map merged with T1 MRI), saggital view, **a** CCA volume, **b** CCM volume, **c** CCP volume

Subsequently, three new masks were created for each patient, each defining one of these volumes (CCA, CCM, CCP). Figure 2 shows these three volumes. Finally, the mean value of FA in each volume was calculated.

### Statistics

For statistical analysis and study design, the institute for epidemiology and biometry at the University of Würzburg was consulted. Statistical analysis was performed with SPSS (IBM, version 27 and GraphPad Prism 10). We calculated a non-parametric two-tailed correlation of patient data, neuropsychological parameters and the mean value of FA in the three parts of the CC. Significance level was chosen at *p* < 0.05, *p* < 0.01 was categorized as highly significant. We then used the Bonferonni method for corrections for multiple comparisons to avoid alpha error accumulation. We also performed a principal component (PC) analysis of the neuropsychological parameters to identify main cognitive domains using GraphPad Prism 10. For each patient, we projected the vector of neuropsychological test results onto the PCs. We then correlated these scores that we obtained for each patient per PC with the FA values in the three areas of the CC using a non-parametric two-tailed correlation. For interpretation, neuropsychological parameters were assigned to a PC on the basis of their loading: a parameter was assigned to the PC with its highest loading, loadings < 0.4 were not taken into account.

Means and standard deviation of each neuropsychological parameter were analyzed for our patient group and a healthy control group using a paired t-test. The control group was matched on the basis of age, gender and educational status.

## Results

### Baseline parameters

28 patients were included in a period of 22 months (02/2017–12/2018) at the University Hospital Würzburg (Table 2). Three patients were excluded due to differing histology or early dismissal. Hence, results are based on 25 patients with a high-grade glioma. 92% of all patients suffered from a WHO °4 glioblastoma, 8% suffered from a WHO °3 astrocytoma. The mean age was 61.7 years. The frontal lobe was infiltrated in 66.7% of all cases, the temporal lobe in 33.3% of all cases. There were only three patients with additional involvement of the parietal or occipital lobe.

**Table 2:** Baseline data

Age	years	61,7 (45–83)
Sex	male female	16 (64.0%) 9 (36.0%)
Histology	Glioblastoma WHO IV Astrocytoma WHO III	23 (92.0%) 2 (8.00%)
Location	Frontal Temporal Parietal Occipital	16 (64.0%) 7 (28.0%) 2 (8.0%) 0 (0.00%)
Side of tumor	Left hemisphere Right hemisphere	11 (44.0%) 14 (56.0%)
Infiltration of the CC	Yes No	7 (28.0%) 18 (72.0%)
Compression or edema of the CC	Yes No	13 (52.0%) 12 (48.0%)

There was a significant association between the patient´s age and FA-values in the truncus of the CC. Higher age was associated with lower FA in this region (*p* = 0.024). In 52% of all patients, there was compression or edema of the CC. Infiltration of the CC was only present in 28% of our patients. Neither compression, nor infiltration of the CC correlated significantly with reduced FA in the CC. Mean FA was highest in the posterior CC, followed by the FA in the anterior CC, FA in the middle CC was the lowest. There were only three patients for which FA in the different parts of the CC did not follow this pattern.

### Outcome

For sake of clarity, significant results of the correlations between FA values in the three parts of the CC and the neuropsychological parameters are shown in Table 3.

**Table 3:** Preoperative correlation between FA values in the CC and neuropsychological parameters, significant results only (p-values and correlation coefficients). Abbreviations: 9-HPT = 9-Hole pegboard test (fine motor skills), SDMT = symbol digit modality test (Graphomotor speed), HVLT = hopkins verbal learning test (verbal memory), ROCF = rey-osterrieth-complex-figure test (visual memory), COWAT = regensburg controlled oral word association test (speech fluency), SCWT = stroop color word test, CCA = anterior CC, CCM, middle CC, CCP = posterior CC, *=significant after Bonferroni correction

			CCA		CCM		CCP	
Test	Parameter	Mean (SD)	p-value	*Correlation coefficient*	p-value	*Correlation coefficient*	p-value	*Correlation coefficient*
9-HPT	Time for sorting in sec.	18 (4.8)	0.440	*−0.162*	0.184	*−0.275*	**0.035**	***−0.423***
SDMT	Number of right answers	17 (6.3)	0.176	*0.279*	0.001 *	***0.641***	**0.014**	***0.484***
HVLT I	Number of right words during run 3	7.2 (2.3)	0.091	*0.345*	**0.018**	***0.468***	0.596	*0.112*
HVLT II	Number of false positive answers, easy level	0.2 (0.4)	0.467	*−0.153*	**0.014**	***−0.485***	0.389	*−0.180*
ROCF I	result	32.2 (4.8)	0.369	*0.188*	**0.034**	***0.426***	0.403	*0.175*
ROCF II	result	12.5 (7.1)	**0.025**	***0.498***	0.061	*0.426*	0.459	*−0.176*
	Time to draw sec	87.8 (43.3)	0.747	*−0.073*	**0.042**	***0.436***	0.891	*0.031*
COWAT	Number of all answers	9.1 (4.4)	**0.011**	***0.508***	0.870	*−0.035*	0.313	*−0.215*
	Number of right answers	8 (4.7)	0.000 *	***0.680***	0.352	*0.199*	0.366	*−0.193*
	Number of wrong answers	1.1 (1.9)	**0.037**	***−0.427***	0.119	*−0.327*	0.913	*−0.023*
SCWT	Part 1 time sec.	9.8 (3.96)	0.901	*−0.026*	0.498	*0.142*	**0.037**	***−0.420***
	Part 3 time sec.	11.2 (8.3)	0.481	*−0.148*	0.404	*−0.174*	**0.010**	***−0.503***

The time needed to sort rods in the **9-HPT** correlated negatively with the FA in the posterior part of the CC (*p* = 0.035). 

The number of correct answers provided in the **SDMT** correlated significantly with a higher FA value in the middle and posterior part of the CC (*p* = 0.001 and 0.014). The amount of right answers during the third run of **HVLT I** correlated significantly with high FA values in the middle part of the CC (*p* = 0.018). In the second part of the test (recognition of words after some time), there is a significant negative correlation between false positive words (mistakes) and FA-values in the posterior CC. The number of all answers in **COWAT** correlated significantly with the FA in the anterior CC. There was a significant correlation between **ROCF I (copy)** and FA-values in the middle CC (*p* = 0.034). The time needed to draw the figure from memory in part II correlated with high FA values in the middle CC (*p* = 0.042). For **JLOT**, there was a significant correlation for 3 of the 20 lines which have to be assigned (*p* = 0.017 and 0.019). Each of them correlated with a different part of the CC. The time needed for part one and three of the **SCWT** (reading of adjectives of colors) correlated negatively with FA-values in the posterior CC (*p* = 0.037 and 0.010).

To avoid a type I error, we performed a Bonferroni correction for the correlation of the neuropsychological parameters with the FA in the three parts of the CC. For 80 neuropsychological parameters and three regions of the CC, this resulted in a significance level of 0.05/240 = 0.0002. The correlations between neuropsychological parameters and FA in one of the regions of the CC, which remain significant according to this conservative correction, are marked with an asterisk in Table 3. Correlations with a significance level between 0.0002 and 0.05 are displayed in bold letters.

Table 4 shows a comparison of the neuropsychological test results of our patients with a healthy cohort matched on the basis of age, gender and educational status. For the sake of clarity, we limited this to the neuropsychological parameters that correlated significantly with FA. Z-scores were determined using the matched healthy control group for each patient, mean z-scores are displayed for significantly correlating parameters.

**Table 4:** This table shows a comparison of the test results of our patients with a healthy cohort matched on the basis of age, gender and educational status. For the sake of clarity, we limited this to the neuropsychological parameters that correlated significantly with FA, Abbreviations: 9-HPT= 9-hole pegboard test, SDMT= symbol digit modality test, HVLT= hopkins verbal learning test, ROCF= rey-osterrieth-complex-figure test, COWAT= regensburg controlled oral word association test, SCWT= stroop color word test

		High grade glioma group (Würzburg) *n* = 25		Healthy control group (Cologne) *n* = 25		Mean z-score (glioma group compared to healthy control)	*p*-value
Test	Parameter	Mean	SD	Mean	*SD*		
9-HPT	Time for sorting in sec.	17.9	4.7	14.3	*3.96*	*0.90*	*0.0014*
SDMT	Number of right answers	17.2	6.3	27.4	*6.97*	*−1.46*	*< 0.0001*
HVLT I	Number of right words during run 3	7.2	2.3	8.84	*2.04*	*−0.80*	*0.007*
HVLT II	Number of false positive answers, easy level	0.2	0.41	0.12	*0.33*	*0.25*	*0.3273*
ROCF I	result	32.2	4.8	46.9	*64.7*	*−1.52*	*0.2639*
ROCF II	result	12.5	7.1	15.8	*7.4*	*−0.4*	*0.1009*
	Time to draw sec	87.8	43.3	86.8	*41.5*	*−0.13*	*0.9171*
COWAT	Number of all answers	9.1	4.4	11.9	*4.1*	*−0.71*	*0.0441*
	Number of right answers	8.0	4.7	10.8	*3.84*	*−0.72*	*0.029*
	Number of wrong answers	1.1	1.9	1.08	*1.94*	*0.04*	*0.4001*
SCWT	Part 1 time sec.	9.8	3.96	7.92	*2.77*	*0.53*	*0.0135*
	Part 3 time sec.	11.2	8.3	5.12	*1.39*	*4.58*	*0.0008*

In the next step, we performed a PC analysis of all neuropsychological parameters. The determination of PCs using parallel analysis, which is the most reliable method, revealed 3 PCs, explaining a cumulative proportion of 52,6% of variance.

For each patient, we projected the vector of neuropsychological test results onto the three PCs. We then correlated these scores that we obtained for each patient per PC with the FA values in the three areas of the CC (Table 5). Correlation between the three PCs and the FA values in the three parts oft he CC provided only one significant correlation – between PC1 and the mean FA in the middle CC (*p* = 0.0235). The neuropsychological parameters were then assigned to the PCs on the basis of their loadings (Table 6). The neuropsychological parameters that mainly contributed to PC1 were DST backwards, number of answers and correct answers in SDMT, time and result of ROCF I, result of ROCF II, all runs of HVLT I, correct answers in HVLT II, time needed for SCWT run 1,2 and 3, mistakes in run 4, time needed for TMT C and D, korrect answers in TMT D, time for sorting out in 9-HPT, right answers in COWAT and time needed for FST.

**Table 5:** Correlation between FA values in the three parts of the Corpus callosum (CC) and the three principle components identified via parallel analysis, abbreviations: PC = principal compnent, CCA = anterior CC, CCM = middle CC, CCP = posterior CC

	PC1		PC2		PC3	
	*p-value*	*Correlation coefficient*	*p-value*	*Correlation coefficient*	*p-value*	*Correlation coefficient*
CCA	0.1865	0.27	0.4273	0.17	0.5504	0.13
CCM	**0.0235**	**0.45**	0.5905	−0.11	0.8466	−0.04
CCP	0.2012	0.26	0.8011	0.05	0.1325	−0.31

**Table 6:** Loadings of all neuropsychological parameters on the three PCs, each parameter is assigned to the PC with the highest loading (grey), loadings < 0.4 are not taken into account, Abbreviations: MMST = mini mental status test, DST = digit span test, 9-HPT = 9-hole pegboard test, SDMT = symbol digit modality test, HVLT = hopkins verbal learning test, ROCF = rey-osterrieth-complex-figure test, COWAT = regensburg controlled oral word association test, SCWT = stroop color word test, JLOT = judgment of line orientation test, FST = find symbol Test, TMT = trail making test

Neuropsychological parameter	PC1	PC2	PC3
MMST	0,71380195	−0,296394	0,05804777
DST forward	0,2151356	0,58158386	−0,0659638
DST backward	0,52706066	−0,0047612	−0,1044009
SDMT correct	0,80574545	0,03289051	−0,3838934
SDMT error	−0,0827387	−0,2191501	0,36733695
SDMT all answers	0,83380722	−0,0525813	−0,2656283
ROCF I time	−0,5705882	−0,3481174	0,13505468
ROCF I result	0,51137732	−0,4637718	−0,385489
HVLT I run 1	0,68492862	0,45064627	0,23244877
HVLT I run 2	0,80572891	0,31868869	0,39414095
HVLT I run 3	0,87300472	0,2129489	0,27953979
HVLT I all	0,84595175	0,34477859	0,32478875
HVLT I scores	0,63904133	0,30280523	0,52470154
SCWT 1 time	−0,509957	0,17150777	0,47487074
SCWT 1 errors	−0,1412306	−0,1051586	−0,2093507
SCWT 2 time	−0,7021229	−0,196268	0,09718695
SCWT 2 errors	−0,3175076	0,17457535	−0,5501174
SCWT 3 time	−0,5697918	0,56018564	0,37643882
SCWT 3 errors	−0,4826116	0,72050547	0,08790457
SCWT 4 time	−0,5704055	−0,3529606	−0,1591961
SCWT 4 errors	−0,709075	−0,3876051	0,06429284
TMT A time	−0,2104882	−0,2973449	0,07991431
TMT B time	−0,4499732	−0,2447907	0,4494489
TMT C time	−0,6001263	−0,4569577	0,13336093
TMT D time	−0,5939353	−0,2971647	0,269735
TMT D error	0,34502358	0,17852579	0,18998175
TMT D correct	0,54163299	−0,3416626	−0,4589225
JLOT left	0,17809064	0,1176555	−0,356329
JLOT right	0,22664869	0,02633915	−0,5898157
JLOT all	0,21508921	0,07888769	−0,5001934
JLOT time	−0,2584489	−0,5414495	0,22733625
9- HPT time sorting in	−0,4697444	−0,1606816	0,46469994
9-HPT time sorting out	−0,6294516	0,27331163	0,47627816
COWAT all	0,45826138	0,56753026	0,07927499
COWAT right	0,52068848	0,40058347	0,19036482
COWAT wrong	−0,2227103	0,43056277	−0,3356598
FST time	−0,5419793	0,09850879	0,15324617
FST left	−0,2306674	0,07742497	−0,4548064
FST right	−0,2752588	0,14957176	−0,3898591
HVLT I run 4 all	0,86138061	−0,184291	0,37474405
HVLT I run 4 score	0,6698837	−0,1756512	0,44772106
HVLT I run 4 time	0,38652943	−0,3799566	0,30594999
HVLT retention (in%)	0,58823897	−0,5313152	0,34061635
HVLT TS run 4	0,43007708	−0,4379074	0,40704506
HVLT II recognition	0,82471179	−0,1802036	−0,2106845
HVLT I easy	−0,3652392	0,61274608	−0,0900446
HVLT II difficult	−0,3376622	0,1646993	0,23779276
ROCFT II result	0,70528952	0,03018	0,16602175
ROCFT II time	−0,0262587	−0,6242119	−0,1453406

## Discussion

### Key results

We found significant correlations between a number of specific neuropsychological parameters and FA-values of different parts of the CC. Remarkably, the direction of correlations did fit our hypotheses (i.e., a lower functional performance went along with lower FA-values).

**Fig. 3:**
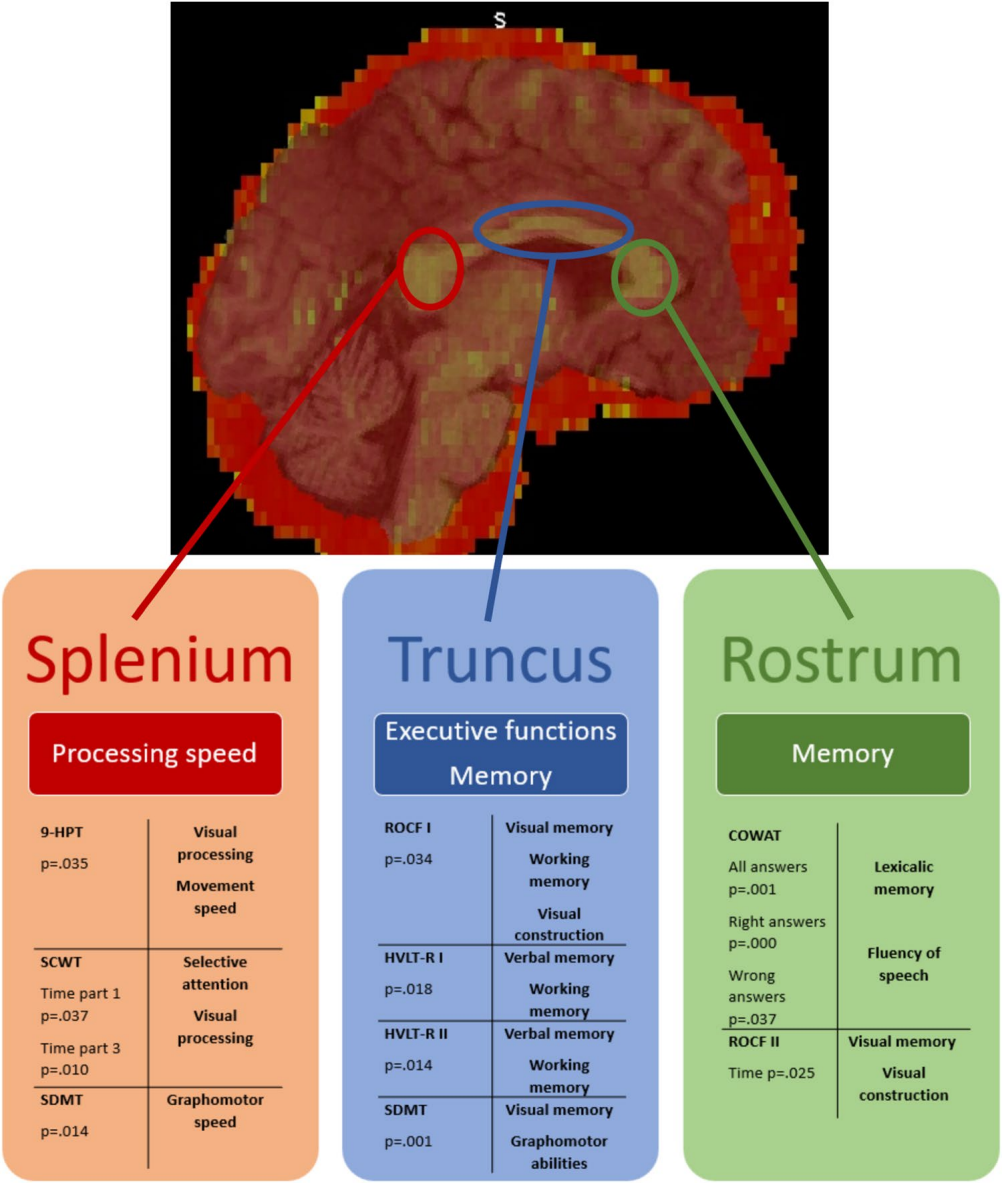
Overview over functions located in the rostrum, truncus and splenium of the CC due to the findings in this study. Abbreviations: 9-HPT= 9-Hole Pegboard Test, SDMT= Symbol Digit Modality Test, HVLT= Hopkins Verbal Learning Test, ROCF= Rey-Osterrieth-Complex-Figure Test, COWAT= Regensburg Controlled Oral Word Association Test, SCWT= Stroop Color Word Test

Figure 3 shows an overview over the main functions associated with FA in the three anatomical regions of the CC in this study due to correlation of the neuropsychological parameters with the three parts of the CC: Lexicalic and visual memory are located in the rostrum, executive functions and memory are located in the truncus and processing speed is located mainly in the splenium. After Bonferroni correction, only two correlations remained significant. The number of neuropsychological parameters is high because we analyzed both the correct and incorrect answers and the processing time for each neuropsychological test of the same cognitive domain, which leads to a strongly conservative multiple comparison correction. Therefore, we would also like to discuss the results with a significance level between 0.002 and 0.05. We consider our results to be particularly valuable for subsequent tests with a higher number of participants.

Two of the neuropsychological tests correlated significantly with WM integrity of the **anterior CC** – the rostrum: **ROCF II** and **COWAT**. Both tests have in common, that they examine memory: visual and lexical memory. The number of correct words named during **COWAT** correlated with higher WM integrity in the rostrum, even after Bonferroni correction. This test examines lexical fluency of speech which is typically impaired in temporo-insular and frontal lesions. Transcallosal fibers of these anatomical regions pass through the anterior part of the CC, which was the site of significant correlation in our patient group [9]. A similar association has been detected for patients suffering from dyslexia: the disease was related to a reduced volume of the anterior CC [24]. Furthermore, reduced FA in the anterior CC was determined in patients with stuttering [13]. For the **ROCF** part II, it was a better quality of the figure drawn from memory, which correlated with higher FA-values in the rostrum. The test checks visual memory and visual constructional ability and is usually associated with lesions in the temporal lobe [36, 42], which anatomically fits the rostrum. There were similar findings for children with severe brain trauma: a reduced FA of the rostrum was associated with worse performance in **ROCF** part I [46].

For the **middle CC** – the truncus –, there were significant correlations of high FA values with **HVLT part I** and **II**,** ROCF part I** and **II** and **SDMT.** All of these tests can evaluate executive functions and working memory. For the **ROCF**, there was a significant correlation between the quality of the drawn figure and WM integrity during initial drawing. Furthermore, patients with higher WM integrity needed more time in part II. This might be explained by an earlier time point of task quitting in patients who were not able to solve the task at all due to a worse neuropsychological state. **HVLT** was related to FA in the truncus as well. For **HVLT** part I it was only the third run of word-repeating, that correlated significantly. Obviously, a difference between patients with higher and lower FA values seems to play a role only after some kind of learning effect has taken place. Furthermore, there was a significant correlation in part II: The significant difference was only there for the easier part of the task. For words similar to the original words and therefore more difficult to keep apart, there was no significant difference between patients with high or low FA, which might be caused by some cognitive decline in all patients due to their disease. A healthy control group and higher number of patients would be necessary to prove this. An association between whole brain WM integrity loss and worse performance in **HVLT** was detected in patients suffering from metabolic syndrome as well. Correlations between **SDMT** and FA- values in the truncus were highly significant, even after Bonferroni correction. Especially the number of right answers correlated, while the number of mistakes did not correlate significantly. This means that patients with worse WM integrity in the truncus did not solve the tasks less careful but were slower concerning processing and graphomotor speed - abilities examined in this test [49, 47]. This test is usually associated with lesions in all supratentorial lobes [47].

**SDMT** was also associated with WM integrity in the **posterior CC** – the splenium, as well as **9-HPT** and **SCWT**. The common component of these tests is processing speed. DSMT analyses graphomotor speed. Since SDMT is usually associated with all brain lobes [47], the correlation with the posterior CC fits the knowledge, that the splenium contains fibers of the parietal, temporal and occipital lobes [11]. Since Symbols have to be matched with numbers in this test it seems likely that visual processing plays an important role as well, concording with the fact, that occipital fibers from the visual cortex pull through the posterior CC [14]. Moreover Gawryluk et al. showed activation of the WM in the posterior CC in functional MRI during SDMT [20]. For the 9-HPT, there was a significant correlation only for sorting the poles in: patients with a high FA in the splenium completed the task faster. This test examines movement speed and is usually used to identify frontal and occipital lesions. Dougherty et al. proved, that occipital-callosal fibers pull through the posterior CC [14], what matches partially with the findings in our study. Sorting the poles out requires less fine motor skills, which might be the reason why there´s no significant relation. A high WM integrity in the posterior CC correlated significantly with fast reading of color adjectives in both levels of difficulty in the **SCWT**. This test usually proves premotor selective attention [30, 44]. However, impaired visual processing might be the cause for the given significant association with the posterior part of the cc that contains occipital transcallosal fibers [14]. The test is usually sensitive especially to frontal but also insular, parietal and occipital lesions [30]. We were not able to show an association with frontal fibers in our study. The fact that occipital and parietal fibers pull through the splenium [11], still explains why **SCWT** is associated with WM integrity in the splenium in our study. Bad results in **SCWT** were described in patients with CC agenesia [12] and a generally reduced WM integrity correlated with results in **SCWT** in patients with geriatric depression [34] as well.

We performed a PC analysis of all neuropsychological parameters in order to substantiate the assignment of cognitive domains to the three areas of CC statistically. The determination of PCs using a parallel analysis, revealed 3 PCs [31]. The neuropsychological parameters were then assigned to the PCs on the basis of their loadings (Table 6). PC1 correlated significantly with the WM integrity of the truncus. Neuropsychological tests assigned to PC1 are DST, SDMT, ROCF I and II, HVLT I and II, time needed for SCWT, TMT, 9-HPT, COWAT and FST. Some of those tests did also correlate with FA values in the trucus in our initial correlation analysis. In addition, the tests that we initially assigned to the splenium and the rostrum are also assigned to PC1. This means that the PC analysis cannot be used to assign the same cognitive domains to the truncus, splenium and rostrum. One of the main reasons is that PC1 explains a large proportion of the variance (30.5%), while PC2 explains only 11.8% and PC3 only 10.3% of the variance. One main reason could be the similar low cognitive baseline of our patients due to the underlying disease. To proove that, a PC analysis of a healthy control group is needed. An analysis of the PCs of neuropsychological functions is planned in a separate study based on larger multi-center population (NOA 19 study) containing glioma patients as well as a healthy control group.

### Complementary findings

In our study group, higher age correlated significantly with lower FA values in the truncus of the CC. A similar connection was shown previously: WM integrity decreases in the aging process [8]. In our study population, age at diagnosis ranged from 45 to 83 years (average 61.7 years), and thereby was slightly under the average of 64 years. If FA should be used as forecast assessment of neurocognitive abilities, this might be an important factor in younger glioma patients.

Mean FA was the highest in the splenium, followed by the rostrum. In the truncus, FA was the lowest. This pattern was already described previously [18]. There was no significant correlation between tumor location, including infiltration and compression of the CC and the FA values in the three parts of the CC.

### Limitations

We used single-center data only for this study to provide uniform neuropsychological und MRI data. Hence, we were able to include 25 patients which leads to limited statistical power allowing to detect only strong correlations at the level of statistical significance. Some of the examined neuropsychological functions did not show a significant association of a microstructural CC lesion and low performance, especially when using correction for multiple comparisons. Hence, the FA in the CC is not sensitive enough to variations of those neuropsychological parameters in this patient group. Therefore, the results of NOA-19 need to be waited for, which will, I.e., examine the relation between the specific locations of the tumor itself with neuropsychological deficits in a larger patient cohort. The neuropsychological tests are similar complex and often difficult to integrate in clinical routine. Furthermore, due to psychological burden of glioma patients, these tests are prone to error: patients waiting for surgery often can´t focus on a test. Patients who were in a poor cognitive state from the beginning are underrepresented in this study since patients with low MMST values (MMST < 20) were excluded, causing selection bias.

## Conclusion

This study confirms that neurocognitive deficits correlate with a reduced microstructural integrity of the CC in patients with high grade gliomas. We were able to link visual and lexical memory to the rostrum, executive functions and working memory to the truncus, and processing speed to the splenium. We consider the FA of the CC for an adequate parameter to examine the influence of distant lesions on neurocognitive abilities. Analyses of a larger patient collective should be executed to corroborate this. Afterwards, further analyses are necessary to examine changes in the FA during adjuvant therapy. This could improve our understanding of tumor therapy toxicity in the future and help to improve the quality of life of high-grade glioma patients.

## Declarations

### Previous presentation of data

The results of this study were presented in advance at the annual meeting of the german society for neurosurgery, parts of the results were published in german language as part of a doctoral thesis by the University of Würzburg.
